# Return to Sport After Anterior Cruciate Ligament Injury: A Scopus-Based Bibliometric Analysis

**DOI:** 10.3390/healthcare14142099

**Published:** 2026-07-14

**Authors:** Nafih Cherappurath, Halil İbrahim Ceylan, Wissem Dhahbi, Muhammed Ali Thoompenthodi, Shamshadali Perumbalath, Masilamani Elayaraja, Mevlüt Yıldız, Libi Kunnel Raveendran, Mohammed Sadique Kozhissery, Jesmy Jose, Raul Ioan Muntean, Sudheesh Chakkummolel Sudhakaran

**Affiliations:** 1 Department of Physical Education, Amal College of Advanced Studies (Autonomous), Nilambur 679329, Kerala, India; 2 Physical Education and Sports Teaching Department, Faculty of Sports Sciences, Atatürk University, Erzurum 25240, Türkiye; 3 Research Unit (UR22JS01) “Sport Sciences, Health and Movement”, High Institute of Sport and Physical Education of Kef, University of Jendouba, Le Kef 7100, Tunisia; 4 Training Department, Police College, Qatar Police Academy, Doha 7157, Qatar; 5 Department of Physical Education, Malabar College of Advanced Studies, Vengara 676519, Kerala, India; 6 School of Economics and Business, RV University, Bengaluru 560059, Karnataka, India; shamshadalip@rvu.edu.in; 7 Department of Physical Education and Sports, Pondicherry University, Puducherry 605014, Puducherry, India; elaya.cricket@gmail.com (M.E.);; 8 Coaching Sciences, Faculty of Sports Sciences, Mugla Sitki Kocman University, Mugla 48000, Türkiye; 9 School of Physical Education and Sports Sciences, Kannur University, Kannur 670567, Kerala, India; libikraveendran@gmail.com; 10 Department of Physical Education, Sacred Heart College (Autonomous), Chalakudy 680307, Kerala, India; jesmyjose@sacredheartcollege.ac.in; 11 Department of Physical Education and Sport, Faculty of Law and Social Sciences, 1 Decembrie 1918 University of Alba Iulia, 510009 Alba Iulia, Romania; 12 Department of Exercise Physiology, Lakshmibai National College of Physical Education, Thiruvananthapuram 695581, Kerala, India; cssudheesh493@gmail.com

**Keywords:** anterior cruciate ligament, return to sport, rehabilitation, athletes, bibliometric analysis, science mapping

## Abstract

**Background:** Returning to sport (RTS) after anterior cruciate ligament (ACL) injury remains a complex challenge in sports medicine, requiring integration of physical recovery, functional performance, and psychological readiness. Although ACL rehabilitation and RTS outcomes have been extensively investigated, the intellectual structure and evolution of this research field have not been comprehensively synthesized. **Objective:** This study aimed to systematically map the global research landscape of ACL injury and RTS using bibliometric analysis. **Methods:** A total of 1368 Scopus-indexed publications published between 1997 and April 2026 were analyzed using performance analysis and science mapping. **Results:** The study identified leading authors, institutions, countries, funding agencies, and journals, and examined collaboration networks, thematic structures, and emerging trends. Scientific output grew substantially after 2012, with accelerated expansion between 2020 and 2025. The United States was the most productive country, and La Trobe University (Australia) the leading institution; the National Institutes of Health (NIH) was the principal funding source. Among 4652 authors, K.E. Webster was the most prolific contributor, and the Orthopaedic Journal of Sports Medicine was the most productive journal. Science mapping showed a shift from surgical and structural perspectives toward multidimensional frameworks emphasizing functional recovery, psychological readiness, neuromuscular performance, injury prevention, and athlete-centered outcomes. Emerging priorities include psychological and neurocognitive readiness, rehabilitation and performance optimization, biomechanical and functional assessment, surgical innovation, outcome validation, and machine learning applications. **Conclusions:** This bibliometric overview offers clinicians, rehabilitation specialists, and researchers an evidence base for guiding future investigation into functional recovery, re-injury risk reduction, and long-term athlete outcomes.

## 1. Introduction

Return to sport (RTS) after an anterior cruciate ligament (ACL) injury and subsequent reconstruction represents one of the most demanding challenges for athletes, both physically and psychologically. Despite advances in surgical techniques and rehabilitation science, the rate of RTS in sport remains lower than expected, with many athletes experiencing diminished performance, shorter careers, and persistent functional limitations [1,2,3]. The high incidence of re-injury exceeding 20% in younger athletes further underscores the vulnerability of this population [3]. Post-anterior cruciate ligament reconstruction, athletes often demonstrate residual functional asymmetries and deficits in dynamic force absorption, reflecting incomplete neuromuscular recovery and an elevated risk of secondary injury [4]. To mitigate these challenges, contemporary rehabilitation emphasizes early, individualized, and criteria-based interventions tailored to each athlete’s physiological and psychological profile, beginning as soon as the day of surgery [5,6].

Although substantial progress has been made in surgical management and rehabilitation, determining an athlete’s readiness to RTS continues to be one of the most complex challenges following ACL injury. RTS is a complex, multifactorial process influenced by biological, psychological, and contextual factors, including injury severity, surgical technique, rehabilitation progress, psychological readiness, and external influences such as competitive demands and stakeholder expectations [7,8,9,10]. Despite growing recognition of these multidimensional factors, there is still no universally accepted framework for RTS decision-making, resulting in considerable variability in clinical practice. Consequently, many clinicians continue to rely on time-based criteria rather than comprehensive, performance-based assessments that incorporate functional capacity, neuromuscular recovery, and sport-specific demands [2,10,11]. Such approaches may increase the likelihood of premature RTS, thereby elevating the risk of secondary ACL injury and compromising the restoration of pre-injury performance levels [1,2]. Furthermore, psychological factors including confidence, fear of re-injury, patient expectations, and motivation, together with external influences from coaches, teams, and sporting organizations, are increasingly recognized as critical determinants of successful RTS. However, these factors remain inadequately integrated into routine clinical assessment and decision-making, highlighting the need for more holistic, evidence-based RTS frameworks [8,9,10].

In response to these challenges, contemporary research has increasingly focused on developing evidence-based frameworks to improve the accuracy and safety of RTS decision-making. Recent models advocate a transparent, shared decision-making process that integrates biological, psychological, and social factors within a biopsychosocial framework [7,9]. Accordingly, objective, criterion-based assessments including muscle strength testing, functional hop tests, and sport-specific performance evaluations are recommended in place of time-based criteria to provide more robust indicators of RTS readiness [10,11]. Emerging approaches, such as functional testing algorithms, dynamic systems theory, and Bayesian network models, further enhance clinical decision-making by incorporating movement variability, neuromuscular function, and multidimensional clinical data to predict RTS outcomes and reinjury risk [4,12].

Modern RTS frameworks advocate a multifactorial, criterion-based approach that integrates physical performance metrics, sport-specific skill restoration, and psychological readiness to ensure safe and sustainable reintegration into competition [3,13]. Structured rehabilitation programs typically progress through clearly defined phases, ranging from impairment resolution and running initiation to agility, plyometric, and sport-specific training, culminating in on-field rehabilitation focused on movement quality, conditioning, and training-load management [14,15]. Progressive strength training remains central to these protocols, promoting neuromuscular control, tissue adaptation, and injury resilience [6,16]. Moreover, addressing psychosocial factors such as confidence, fear of re-injury, and motivation through a biopsychosocial framework has been shown to enhance rehabilitation adherence and RTS success [3,13,17]. A pivotal catalyst for this conceptual shift was the 2016 Bern consensus statement, which formalized criteria-based, multidimensional return-to-sport decision-making and explicitly incorporated psychological readiness alongside physical recovery [18]. This consensus, together with subsequent validation of objective discharge criteria [6,19], has shaped much of the rehabilitation and return-to-sport literature published over the past decade. Therefore, a comprehensive, dynamically monitored rehabilitation process grounded in objective RTS testing and athlete feedback remains critical for optimizing outcomes and minimizing the risk of re-injury in athletes following ACL reconstruction [17,20]. Throughout this article, ‘return to sport’ (RTS) denotes full reinstatement to competitive athletic participation, consistent with the criterion-based framework described by Ardern et al. [21]; “return to play” (RTP) refers to the broader resumption continuum including training reintegration, and both terms are retained where the source literature does not apply the distinction.

Scholars have examined ACL injuries and RTS both as distinct research areas and in combination to understand their interrelated dynamics. Many studies have extensively explored the complex landscape of ACL injuries [21,22,23,24] and RTS [25,26,27,28,29,30,31]. Some studies have integrated both domains to provide a comprehensive landscape, particularly those employing systematic literature reviews, meta-analyses, and scoping reviews [27,28,32,33,34,35]. Prior bibliometric investigations in this domain have examined ACL reconstruction techniques [24,36,37,38], surgical augmentation procedures [39,40], or pediatric ACL injury [41] as discrete topics. To our knowledge, no prior study has integrated ACL injury with RTS outcomes as a combined bibliometric axis, applied dual-platform science mapping (VOSviewer and Biblioshiny/R) across a dataset extending to April 2026, or produced thematic evolution analysis with explicit temporal cut-points for this domain.

The present study addresses this gap by providing a comprehensive Scopus-based bibliometric analysis of RTS research following ACL injury. A rigorous methodology was adopted, including multi-stage screening with predefined inclusion and exclusion criteria, inter-rater reliability assessment, and thesaurus-standardized keyword mapping. Performance analysis was conducted to evaluate publication trends, influential authors, institutions, countries, funding agencies, journals, and highly cited publications. In addition, science mapping techniques, including co-occurrence analysis, bibliometric coupling, thematic evolution, trend-topic analysis, and overlay visualization, were employed to characterize the intellectual structure of the field. These analyses identify emerging research themes, reveal collaboration patterns, and highlight future research priorities in this rapidly evolving area of sports rehabilitation.

## 2. Methods

The Section 2 outlines the bibliometric approach, encompassing keyword selection, database selection, data handling, methodological design, and the analytical tools employed. Bibliometric analysis utilizes statistical methods to examine published literature and bibliographic data, enabling researchers to track disciplinary growth, identify emerging trends, and evaluate research performance and collaboration [42,43]. Its cross-disciplinary nature and scalability have made it widely used. Adopting the framework of Zupic and Čater [44], this study follows a systematic workflow of design, data retrieval, analysis, visualization, and interpretation.

The study is designed to map and forecast emerging research areas on ACL injuries and RTS. Accordingly, search terms were refined around two core themes: ‘ACL injury’ and ‘return to sport’. The search keywords were selected from relevant sources, including ACL [36,39,45,46,47,48] and RTS [27,28,30,31]. Before keyword co-occurrence analysis, a standardized thesaurus file was constructed to merge orthographic variants, singular/plural forms, and accepted abbreviations of core terms into canonical representations (e.g., ‘return to sport’, ‘ACL reconstruction’, ‘patient-reported outcome measures’). This thesaurus was applied in both VOSviewer (v.1.6.20) [49] and Biblioshiny (R-package bibliometrix v.5.4.1) [43], which were selected because they jointly enable performance analysis and multiple complementary science-mapping techniques (keyword co-occurrence, thematic mapping, bibliographic coupling, and thematic evolution) within a single, reproducible, freely available workflow, consistent with established bibliometric methodology guidance [44,50]. Co-citation analysis was not performed separately, since bibliographic coupling and keyword-based mapping were judged most informative for characterizing the conceptual rather than purely citation-based structure of this literature; this represents a methodological choice rather than an omission, and is noted as a direction for future complementary analysis. Alternative expressions for ‘ACL’ and ‘return to sport’, along with the complete search terms and search query, are provided in Supplementary Materials. TITLE-KEY ((“anterior cruciate ligament” OR “ACL”) AND (“return to play*” OR “return-to-sport*” OR “return to sport*” OR “back to sport*” OR “return-to-competition*” OR “return to competition*”)): This keyword set ensures comprehensive and accurate retrieval of relevant studies, encompassing diverse research areas while minimizing the risk of omitting pertinent literature.

After finalizing the keywords, the next step is to choose a database and develop the search strategy. While previous studies have used Web of Science [24,46,47,48] and Scopus [41,51]. This study employs Scopus due to its far-reaching coverage, multidisciplinary source inclusion, and robust citation indexing [50,52]. Scopus is widely regarded as a leading database for bibliometric analysis, offering broader journal coverage than the Web of Science, particularly in the humanities and social sciences, along with advanced citation mapping, detailed author and affiliation profiles, and a diverse range of document types [53,54,55,56,57]. With over 76 million records and high citation accuracy, Scopus provides comprehensive and reliable coverage, making it a preferred source for bibliometric research and evaluation [58,59,60].

**Figure 1:**
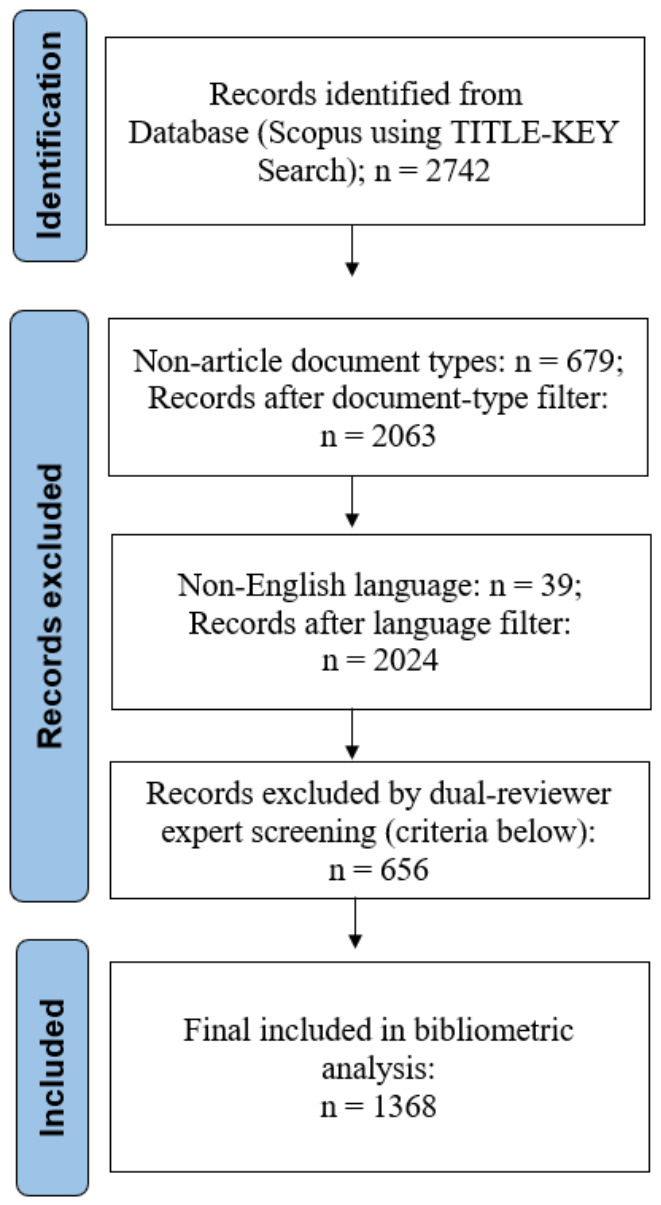
Bibliometric data collection workflow (PRISMA).

Our search strategy begins with keywords on ‘article title’, given its critical role in scientific literature [61,62]. Recognizing that limiting the search to article titles could exclude relevant studies, the query was expanded to titles and indexed Scopus keywords (TITLE-KEY) to increase retrieval sensitivity. The TITLE-KEY search retrieved 2742 records from Scopus. Filtering for peer-reviewed journal articles (excluding editorials, conference papers, letters, and book chapters) removed 679 records, yielding 2063 documents. Subsequent restriction to English-language publications removed 39 records, yielding 2024 documents. Two independent reviewers then screened all 2024 records against the predefined inclusion and exclusion criteria (see below); 656 articles were excluded, and disagreements were resolved by consensus. The final dataset comprises 1368 English-language journal articles published between 1997 and April 2026. Figure 1 presents the modified PRISMA-compliant flowchart. Although PRISMA was originally developed for systematic reviews of intervention effectiveness, its flow-diagram structure was adapted here solely to document the multi-stage identification, screening, and inclusion process transparently, consistent with increasing use of PRISMA-style flow diagrams in bibliometric studies to standardize reporting of record attrition [50]; no claim of systematic-review methodology (e.g., risk-of-bias assessment, evidence synthesis) is implied.

Two reviewers (N.C. and S.P.) independently screened titles and abstracts of all 2024 records against the predefined inclusion and exclusion criteria (see below), with full-text review conducted for equivocal records; 656 articles were excluded, and disagreements were resolved through consensus discussion. Inter-rater reliability was calculated using Cohen’s Kappa (κ = 0.85). Eligibility screening therefore combined metadata-level assessment with targeted full-text verification, whereas the subsequent bibliometric and science-mapping analyses (performance analysis, keyword co-occurrence, thematic mapping) were conducted exclusively on Scopus-indexed metadata (titles, abstracts, author keywords, citation, and affiliation data) for the 1368 included records. Full-text inaccessibility was applied as an exclusion criterion because confirming RTS/RTP outcome reporting and human-participant status, both required for eligibility, was not reliably possible from metadata alone for a subset of equivocal records.

Inclusion criteria were: (i) peer-reviewed original research or systematic review/meta-analysis articles; (ii) published in English; (iii) indexed in Scopus; (iv) reporting data on ACL injury, ACL reconstruction, or associated rehabilitation with explicit reference to RTS or RTP outcomes in human participants. Exclusion criteria were: (i) non-human studies; (ii) case reports or letters; (iii) studies addressing ligament injuries other than ACL as the primary focus; (iv) articles where full-text retrieval was not possible, precluding confirmation of inclusion criteria (i) and (iv) above.

## 3. Results

The Section 3 is divided into two primary components: performance analysis and science mapping analysis. The performance analysis evaluates the annual scientific output across several dimensions, including contributing countries and institutions, funding agencies, high-impact journals, influential authors, and key publications in the field of RTS after ACL injury research. It also investigates patterns of international collaboration at both institutional and national levels. The science mapping analysis employs bibliometric techniques to uncover the major research themes, intellectual structures, and emerging trends within the domain. This approach further facilitates the identification of knowledge gaps and the formulation of prospective research directions related to RTS following ACL injury.

### 3.1. Performance Analysis

This bibliometric analysis encompasses publications from 1997 to 2026, yielding a total of 1368 documents distributed across 206 journal sources. The field demonstrates robust, sustained growth, with an annual increase in publication rate of 15.54%, highlighting escalating scholarly interest in RTS research following ACL injury. The average document age of 4.97 years indicates that the research area is both active and rapidly advancing. On average, each publication has received 29.68 citations, reflecting a moderate to high academic impact. Collectively, the 40,274 references cited across these publications underscore the field’s extensive and diverse theoretical foundation. The dataset includes 3144 Keywords Plus (ID) and 1795 Author Keywords (DE), suggesting a broad thematic scope and multidisciplinary engagement within this research domain. Authorship analysis identifies 4652 unique contributors. Moreover, an international co-authorship rate of 25% demonstrates substantial cross-national collaboration, underscoring the global relevance and interconnected nature of research on RTS after ACL injury.

#### 3.1.1. Annual Scientific Publication

**Figure 2:**
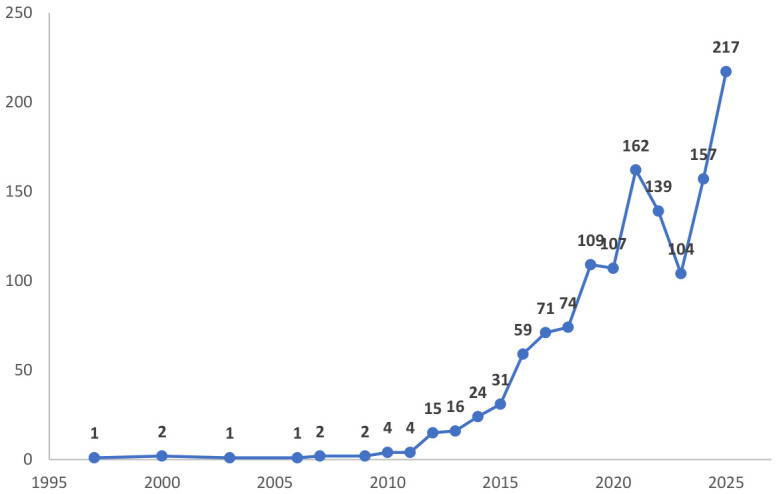
Annual scientific publication output (1997 to April 2026), *n* = 1368.

The publication trend from 2012 to 2025 shows a consistent, progressive increase in research activity in the field. The highest number of publications was observed in 2025 (*n* = 217), followed by 2021 (*n* = 162) and 2024 (*n* = 157). In contrast, the period between 1997 and 2011 exhibited relatively low productivity, with only 1 to 4 publications per year and minor fluctuations. A marked upward trajectory began in 2012, when publication numbers rose to 15, signaling sustained research growth. This expansion continued steadily throughout the subsequent years. Notably, the period between 2020 and 2025 shows an accelerated surge in scholarly output, accounting for the majority of the dataset’s total publications. This sharp rise reflects an increasing academic focus and research commitment toward understanding and improving RTS outcomes following ACL injury (Figure 2).

#### 3.1.2. Country-Wise Contribution

**Figure 3:**
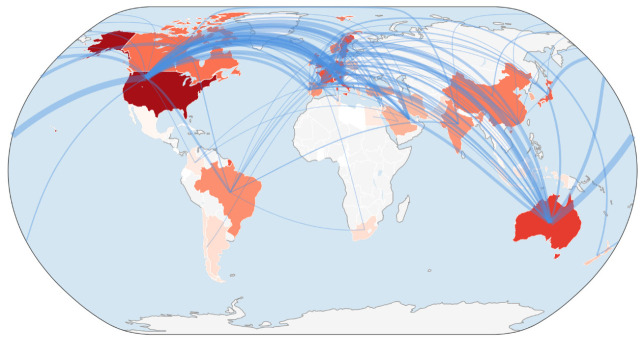
Country collaboration map.

Country-level analysis highlights a robust global research network in RTS after ACL injury, with active contributions from institutions across multiple continents. The United States overwhelmingly dominates the field, accounting for 560 publications, reflecting its longstanding leadership and research capacity in sports medicine and rehabilitation. Australia follows with 138 publications, while Italy (109), the United Kingdom (91), and Germany (89) also make substantial contributions, underscoring the strong presence of Western research institutions. Within Europe, Sweden (98) and France (90) exhibit notable productivity, further demonstrating the region’s consistent engagement in this area. In Asia, Japan (68) and China (35) have emerged as key contributors, signifying a growing emphasis on ACL rehabilitation and RTS research in the region. Additional contributions from Canada (51), the Netherlands (49), Norway (42), Switzerland (39), Brazil (31), and reinforce the global and collaborative nature of this research field (Figure 3).

**Figure 4:**
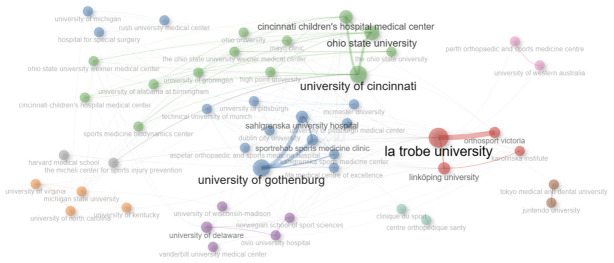
Institution collaboration map. Method Parameters: Network Layout: Automatic layout, Clustering Algorithm: Louvain, Normalization Method: Association, Network Size: Number of Nodes: 50, Repulsion Force: 0.5.

Figure 4 shows the institutional collaboration network, highlighting distinct clusters and linkages among key research organizations involved in RTS studies after ACL injury. Institutional affiliation data reveal that La Trobe University (Australia) leads the field with 70 publications, underscoring its central role in advancing rehabilitation and sports medicine research. Prominent collaborative clusters include Cincinnati Children’s Hospital Medical Center (USA) and The Ohio State University (USA), both of which contribute significantly to the scientific output and network connectivity. In terms of research funding, the National Institutes of Health (NIH, USA) is the primary funding agency, supporting 62 publications in this domain. Additional major sponsors include the National Institute of Arthritis and Musculoskeletal and Skin Diseases (USA), Arthrex (USA), Centrum för Idrottsforskning (Sweden), and the National Institute of Child Health and Human Development (USA).

#### 3.1.3. Most Published Journals

Table 1 presents the top 10 journals contributing to research on RTS after ACL injury, highlighting their total publications, publishers, citation metrics, and impact indicators. The Orthopaedic Journal of Sports Medicine ranks first with 175 publications, underscoring its prominent role as a key dissemination platform for clinical and rehabilitation research on ACL injuries. It is followed by Knee Surgery, Sports Traumatology, Arthroscopy (137 publications), and the American Journal of Sports Medicine (120 publications), both of which are long-established and highly reputable outlets in the field.

#### 3.1.4. Most Influential Publications

Table 2 presents the ten most highly cited publications in RTS after ACL injury, as indexed in Scopus up to April 2026. Citation frequency serves as a proxy for scholarly influence, reflecting the enduring impact of these studies on clinical practice and research development in ACL rehabilitation and sports medicine. Citation counts (Table 2) reflect total Scopus-indexed citations as of April 2026 and were not adjusted for self-citation, consistent with standard Scopus citation reporting; self-citations were therefore included in all citation-based metrics reported in this study.

#### 3.1.5. Most Prolific and Influential Authors

Table 3 presents the leading authors in RTS after ACL injury research, ranked by both total publications (TP). The analysis highlights a concentration of scholarly influence among a small group of highly productive researchers who have shaped the field’s development through extensive empirical and clinical contributions.

### 3.2. Science Mapping

**Figure 5:**
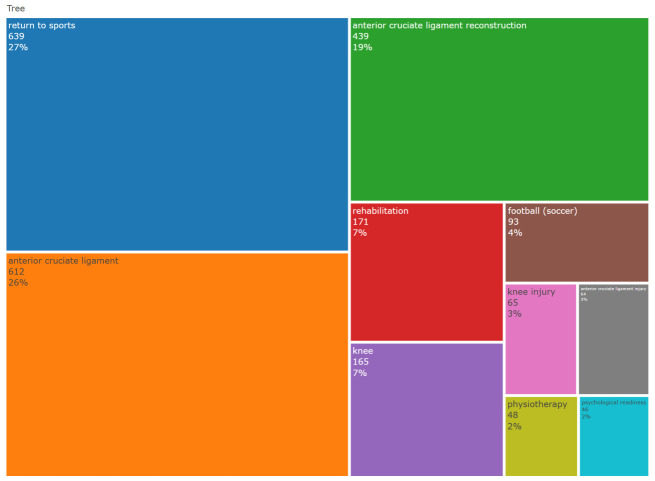
Word tree map.

**Figure 6:**
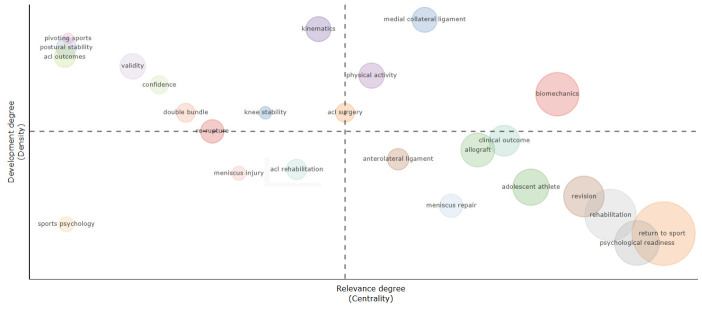
Thematic map. Field: Author’s Keywords, Parameters: Number of Words: 250, Min Cluster Frequency (per thousand docs): 5, Number of Labels: 1, Label size: 0.5, Community Repulsion: 0, Clustering Algorithm: Walktrap.

**Figure 7:**
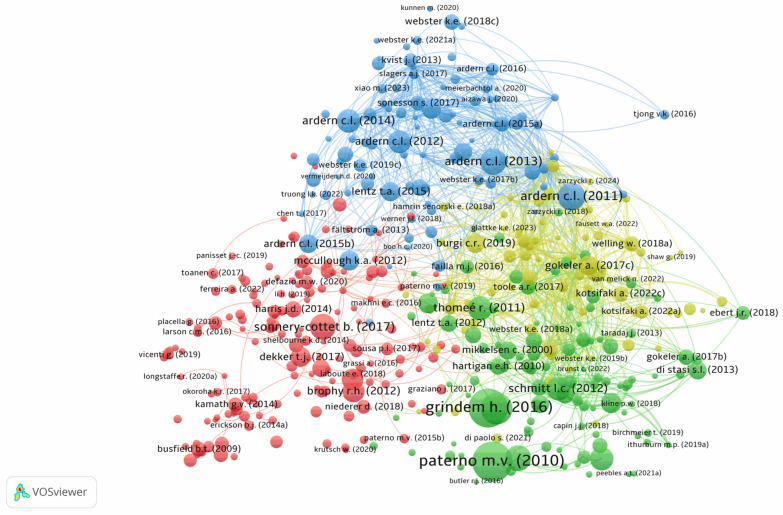
Bibliometric coupling network analysis. Minimum citation count per document: 20; documents meeting threshold: 479 of 1368.

**Figure 8:**
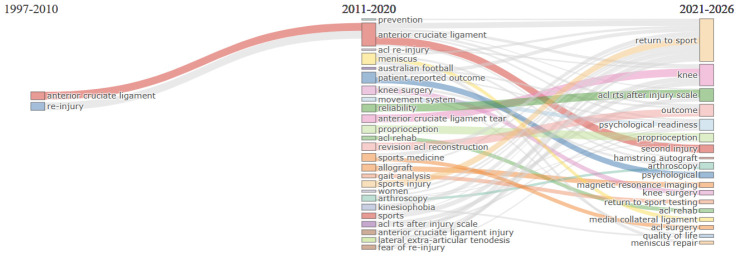
Thematic evolution map. Parameters: Number of words: 250; min cluster frequency (per thousand docs): 5; weight index: inclusion index weighted by word occurrences; number of cutting points: 2 (Cutting Year 1: 2010, Cutting Year 2: 2020); clustering algorithm: Walktrap.

**Figure 9:**
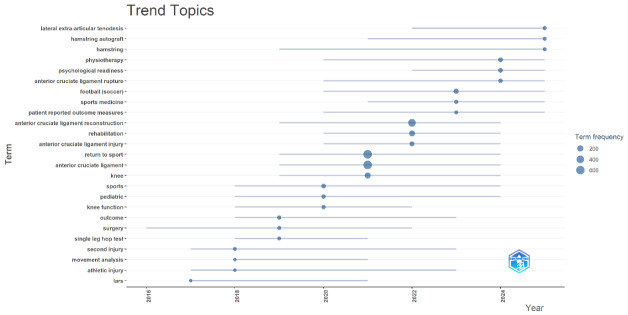
Trend topics. Field: author’s keywords; word minimum frequency: 5, number of words per year: 3.

**Figure 10:**
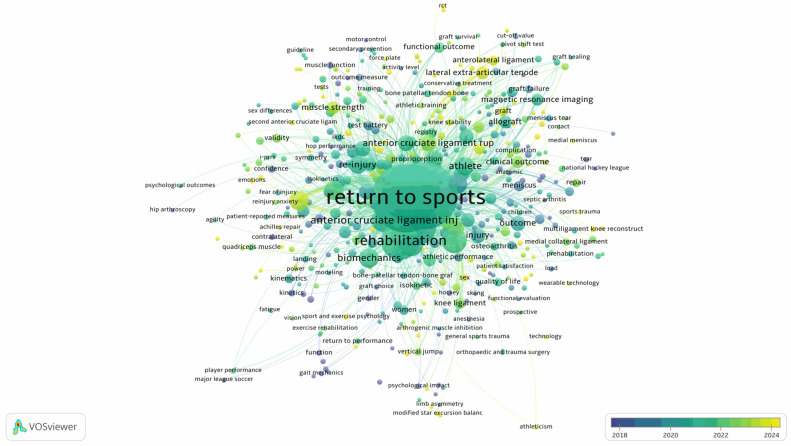
Co-occurrence (overlay) visualization map. Minimum number of keyword occurrences: 2, no. of keywords met threshold: 463, total number of keywords: 1554.

This section presents a systematic visualization of the scientific landscape of ACL injury and RTS research across athletic populations, examining prevailing research patterns and prospective directions. The science mapping analysis is structured into two major components: (i) thematic concentration, which employs a tree map (Figure 5), a thematic map (Figure 6), and bibliometric coupling (Figure 7) to delineate the principal research domains and conceptual linkages within the field; and (ii) emerging trends and future directions, illustrated through a thematic evolution map (Figure 8), trending keywords analysis (Figure 9), and a keyword overlay visualization (Figure 10), providing insights into the dynamic progression of research themes and identifying promising avenues for further investigation.

A visualization of the articles’ most common keywords appears before we implement science mapping techniques (Figure 5). The science mapping techniques build their analysis units from these fundamental keywords. A Word TreeMap from Authors’ Keywords (DE) in Biblioshiny highlights the most prominent terms, with return to sport (639, 25%) and anterior cruciate ligament (612, 24%) being the most frequent, followed by anterior cruciate ligament reconstruction (439, 17%), rehabilitation (171, 7%), knee (165, 6%), football (soccer) (93, 4%), knee injury (65, 3%), anterior cruciate ligament injury (64, 3%), anterior cruciate ligament-return to sport after injury scale (47, 2%) and physiotherapy (43, 2%).

#### 3.2.1. Thematic Concentration

We applied a thematic map (Figure 6), to identify and describe the primary themes, patterns, and emerging areas within the research topic. The identified keywords are presented in Table 4.

The bibliometric coupling network (Figure 7) reveals four major thematic clusters related to RTS research following ACL injury. The blue cluster centers on RTS readiness, emphasizing objective assessment criteria such as limb symmetry, muscle strength, hop performance, gait mechanics, and landing strategies. The green cluster underscores the importance of restoring strength, symmetry, and neuromuscular control as prerequisites for the safe resumption of sport. The yellow cluster highlights psychological readiness, movement quality, and the role of individualized rehabilitation approaches. Finally, the red cluster focuses on the risk of reinjury, surgical technique selection, and population-specific factors influencing rehabilitation outcomes.

#### 3.2.2. Thematic Evolution and Future Directions

Three visualization maps were employed to trace thematic development and identify emerging research directions on ACL injuries in athletes. A thematic evolution map (Figure 8) generated in Biblioshiny using author keywords, with cut points at 2010 and 2020, highlights recent advances in the field. Figure 9 displays the most prominent keywords, while Figure 10 presents a VOSviewer co-occurrence overlay that maps relationships among the keywords.

Figure 8 demonstrates the progressive evolution of ACL injury research across three periods. From 1997 to 2010, the focus centered on the anterior cruciate ligament and reinjury. Between 2011 and 2020, themes expanded into a more diverse thematic structure, with emerging keywords such as ACL, meniscus, patient report outcome, knee surgery, reliability, revision ACL reconstruction, sports medicine, ACL tear, allograft, sports injury, arthroscopy, kinesiophobia, sports, ACL-RTS after injury scale, ACL injury, lateral extra-articular tenodesis, fear of re-injury, gait analysis, movement system, ACL rehabilitation, women, Australian football, prevention, and ACL re-injury. In 2021–2026, ‘return to sport’ remains central but connects with a broader network of keywords such as knee, ACL-RTS after injury scale, outcome, psychological readiness, proprioception, second injury, arthroscopy, psychological, magnetic resonance imaging, knee surgery, ACL rehabilitation, return to sport testing, medial collateral ligament, ACL surgery, quality of life, meniscus repair, hamstring autograft, physiotherapy, pediatric and fear of re-injury.

Figure 9 presents the evolution of major research keywords related to RTS studies after ACL injury (Table 5). This trend analysis highlights a shift from structural and surgical emphases toward integrative, athlete-centered paradigms that blend physiological, biomechanical, and psychological factors to improve RTS outcomes and reduce the risk of reinjury.

The overlay visualization (Figure 10) highlights emerging keywords in yellow, reflecting the latest trends in RTS research following ACL injury. Recent keywords include psychometric properties, back in action, badminton, brace, emotion, physical function, growth plates, neuromuscular, hamstrings muscle, ground reaction force, drop jump, quadriceps tendon graft, modified star excursion balance, reactive strength index, athletes, technology, patellar ligament, technology, wearable technology, neurocognitive, return to sport criteria, recovery, postoperative outcomes, quadriceps autograft, multi-ligament knee reconstruction, knee dislocation, sports trauma, ramp lesion, medical meniscus, quadriceps tendon autograft, let, peroneus longus, anterolateral rotatory instability, propensity score, lateral tenodesis, revision anterior cruciate ligament, functional capacity, pre-injury level, meniscus injury, meniscectomy, force plate, blood flow restriction training, rehabilitation outcomes, isokinetic evaluation, second knee injury, contralateral rupture, second ACL injury, RCT, telerehabilitation, reoperation, reactive strength index, psychological readiness, electromyography, motor learning, rupture, sports physiotherapy and neuromuscular training.

## 4. Discussion

This bibliometric analysis provides a comprehensive assessment of the evolution and current landscape of research on RTS following ACL injury. Drawing on 1368 Scopus-indexed publications spanning 1997 to April 2026, the study identifies leading authors, institutions, funding agencies, influential publications, and emerging thematic trends. Its objective is to inform scholars and practitioners by delineating key contributions, prevailing research priorities, and prospective directions for future inquiry. The analysis reveals a marked increase in research output beginning around 2012, reflecting a growing academic and clinical commitment to understanding RTS processes after ACL injury. This acceleration coincides with the publication of consensus-based, criteria-driven RTS frameworks, most notably the 2016 Bern consensus statement [18], which formalized psychological readiness and objective discharge criteria as core RTS components; the thematic evolution data in this study (Figure 8, Table 5) corroborate this shift, showing psychological and criteria-based keywords emerging as dominant themes from 2016 onward. From 2015 onward, the field experienced rapid expansion, with a particularly sharp rise in publications between 2020 and 2025. This acceleration signifies the field’s transition toward maturity and underscores a global shift toward evidence-based, multidisciplinary rehabilitation and RTS frameworks designed to optimize functional recovery and minimize the risk of reinjury.

The analysis further reveals a broad international distribution of research, with the United States emerging as the leading contributor in total publications, consistent with its sustained federal research infrastructure (NIH funding of 62 publications) and the high volume of collegiate and professional sports medicine programs generating ACL caseloads. Australia’s strong representation reflects the concentrated influence of a small number of highly productive research groups, particularly at La Trobe University, rather than broad national output. At the institutional level, La Trobe University (Australia) holds a central position in advancing research on RTS following ACL injury, while the National Institutes of Health (NIH, USA) is the primary funding source for this field. Among the 4652 identified authors, Webster, K.E., is recognized as the most prolific contributor and also achieves the highest fractionalized authorship score, underscoring her sustained scholarly impact in ACL rehabilitation research. Additionally, we examined the publication patterns of journals in this field. The Orthopaedic Journal of Sports Medicine ranks as the most productive journal, serving as a leading platform for disseminating clinical, biomechanical, and rehabilitation-oriented studies on ACL injury and RTS outcomes.

### 4.1. Research Hotspots and Trends

The recent evolution of research on RTS following ACL injury has revealed a clear shift toward multidimensional, data-driven recovery frameworks. This period marks the consolidation of biomechanical, psychological, surgical, and technological domains into an integrated understanding of post-ACL rehabilitation. The thematic synthesis reveals six interrelated research clusters that collectively redefine how successful RTS is conceptualized and achieved (Table 6). The findings below synthesize what the underlying primary studies captured in this corpus report, as reflected by recurring keyword and citation patterns; they describe where research attention has been concentrated rather than constituting independent bibliometric validation of clinical effectiveness.

A dominant trend across recent studies involves psychological and cognitive determinants of RTS. Keywords such as psychological readiness, confidence, fear of re-injury, and emotions reflect growing recognition that recovery extends beyond physical healing, with mental resilience, motivation, and confidence reported to influence rehabilitation adherence and successful return [72,73,74,75,76,77,78,79]. The emergence of neurocognitive training reflects growing interest in decision-making, reaction time, and perceptual-motor coordination as components of on-field performance and injury prevention [80,81], aligning RTS research with biopsychosocial models that integrate emotional and cognitive recovery with physical readiness. Recent studies emphasize advanced rehabilitation strategies designed to enhance recovery efficiency and performance outcomes. Blood flow restriction training has gained traction as a time-efficient modality for maintaining muscle hypertrophy and strength under lower mechanical loads during early rehabilitation [82,83], while integration of physiotherapy, proprioceptive retraining, and isokinetic evaluation reflects a multidimensional approach to restoring functional capacity [84,85,86]. Rehabilitation and performance optimization are increasingly treated as overlapping rather than sequential processes, with neuromuscular control supporting both athletic performance and long-term knee health.

Restoring functional symmetry and neuromuscular efficiency is an increasingly emphasized domain. Keywords such as gait symmetry, limb asymmetry, quadriceps femoris strength, and ground reaction force reflect growing reliance on quantitative performance metrics for evaluating readiness for sport resumption [87,88,89,90,91,92], marking a methodological shift from subjective clinician assessment toward objective, performance-based indicators [93]. Countermovement jump and isokinetic testing provide measurable insight into lower-limb power, balance, and kinetic-chain efficiency associated with re-injury risk reduction [84,87,91,92]. Refined surgical techniques and graft selection remain key determinants of long-term outcomes. The increasing prominence of hamstring and quadriceps autografts, anterolateral ligament reconstruction, and lateral extra-articular tenodesis reflects ongoing debate regarding optimal rotational stability, while growing attention to multi-ligament reconstruction and meniscus-preserving approaches signals a shift toward comprehensive joint preservation. These innovations depend on integration with personalized rehabilitation and neuromuscular retraining to translate into functional benefit [94,95,96,97].

Growing emphasis on clinical and patient-reported outcome measures (PROMs) reflects an ongoing effort to standardize RTS success criteria [98,99,100], bridging subjective recovery perceptions with objective functional data. Keywords such as validity and patient-accepted symptom status indicate a shift toward outcome frameworks that are psychometrically sound and patient-centered. However, cross-study comparability and universal RTS benchmarks remain unresolved. Technological integration represents an emerging frontier in RTS research. Magnetic resonance imaging, machine learning, and AI-based predictive analytics reflect the field’s transition toward precision rehabilitation [101,102], facilitating early detection of structural abnormalities, individualized progress tracking, and risk stratification for second ACL injury [103,104]. Machine learning further enables data-driven personalization of rehabilitation protocols based on athlete-specific biomechanical and psychosocial profiles [105].

### 4.2. Practical Implications

The findings of this bibliometric analysis provide several clinically relevant implications for sports physicians, orthopedic surgeons, physiotherapists, strength and conditioning specialists, and other professionals involved in ACL rehabilitation and RTS decision-making. These implications derive from documented shifts in research emphasis rather than from direct evaluation of intervention efficacy, since bibliometric methods characterize publication patterns and thematic structure rather than clinical outcomes; they are intended to inform awareness of evolving evidence priorities rather than to constitute treatment recommendations. First, the evolution of the literature demonstrates a clear transition from time-based and surgery-centered approaches toward multidimensional, criteria-based RTS models. Consequently, the literature suggests that RTS decisions may benefit from incorporating objective measures of functional recovery, including limb symmetry indices, quadriceps and hamstring strength assessments, hop-test performance, movement-quality analysis, and neuromuscular control evaluations, rather than relying solely on postoperative timelines. Second, the increasing prominence of psychological readiness highlights the growing importance of integrating patient-reported outcome measures (PROMs) and validated psychological assessment tools into routine rehabilitation practice. Fear of re-injury, confidence, motivation, and psychological readiness have emerged as key determinants of successful RTS and may be considered alongside physical performance outcomes before clearance is granted. Third, the growing emphasis on graft selection, surgical techniques, and re-injury risk supports the implementation of individualized rehabilitation pathways. The literature indicates that individualized rehabilitation pathways may be beneficial, taking into account surgical characteristics, injury history, sport-specific demands, and athlete-specific risk profiles to optimize recovery and reduce the likelihood of secondary ACL injury. Fourth, emerging trends in machine learning, motion analysis, wearable technologies, MRI-based assessment, and isokinetic testing indicate a movement toward precision rehabilitation. These technologies can facilitate longitudinal monitoring of recovery, identify residual biomechanical deficits, and support evidence-based RTS decisions through objective and individualized performance tracking. Finally, the convergence of biomechanical, psychological, surgical, and technological research domains reinforces the importance of interdisciplinary collaboration. Effective RTS management requires coordinated input from orthopedic surgeons, sports physicians, physiotherapists, sports psychologists, biomechanists, and performance specialists. Collectively, these findings highlight the growing emphasis on standardized, athlete-centered, and evidence-informed rehabilitation frameworks aimed at maximizing functional recovery, minimizing reinjury risk, and promoting sustainable long-term athletic performance following ACL injury.

### 4.3. Limitations and Future Directions

While this study offers valuable insights into the global research landscape on ACL injury and RTS, several limitations should be acknowledged. First, relying solely on the Scopus database may have caused the omission of relevant studies indexed elsewhere, such as Web of Science or PubMed. Although Scopus is well-regarded for its extensive journal coverage, citation indexing, and suitability for bibliometric analysis, depending on just one database can introduce potential selection bias. PubMed/MEDLINE indexes additional clinical and rehabilitation-focused biomedical journals not covered by Scopus and does not provide citation counts, so studies retrievable only through MEDLINE were necessarily excluded from this analysis, and citation-based rankings (Table 2) reflect Scopus indexing only. Web of Science applies distinct journal-inclusion criteria and a separate citation index, which can shift country, institution, and author productivity rankings relative to Scopus [55,57]. Consequently, the leading-contributor findings reported here should be interpreted as Scopus-specific rather than as a universally invariant ranking of the field. Future studies might benefit from comparing multiple databases to strengthen findings and verify the stability of bibliometric indicators across platforms. Second, using keyword-based search strategies may have restricted the retrieval of studies that use different terminology or emerging concepts not included in the predefined search criteria. Despite the multi-stage screening process and expert validation, bibliometric methods inherently depend on the accuracy and consistency of metadata from indexed publications. Third, bibliometric analysis examines patterns of scientific production, collaboration, and thematic development but does not directly evaluate the methodological quality or clinical effectiveness of individual studies. Therefore, while this approach provides a broad understanding of research trends and knowledge structures, it should complement systematic reviews and meta-analyses rather than replace clinical evidence synthesis. Future research should consider incorporating multiple databases, applying advanced text-mining or natural language processing techniques to improve thematic accuracy, and combining bibliometric mapping with qualitative or mixed-method approaches. Such integration could lead to a more comprehensive understanding of both the intellectual framework and practical implications of ACL rehabilitation and RTS research.

## 5. Conclusions

This bibliometric analysis provides a comprehensive overview of the global scientific landscape of return-to-sport (RTS) research following anterior cruciate ligament (ACL) injury. Based on 1368 Scopus-indexed publications spanning nearly three decades, the findings demonstrate substantial growth in research activity, particularly since 2012, reflecting the increasing clinical and scientific importance of optimizing rehabilitation and RTS outcomes in athletic populations. The United States, La Trobe University, the National Institutes of Health, and leading researchers such as K.E. Webster have played pivotal roles in shaping the field’s development.

Thematic and science-mapping analyses revealed a clear evolution from predominantly surgical and structural perspectives toward multidimensional rehabilitation frameworks integrating functional performance, psychological readiness, neuromuscular recovery, injury prevention, and emerging technological approaches. Recent research trends increasingly emphasize criteria-based RTS decision-making, personalized rehabilitation strategies, objective functional assessment, and advanced monitoring and analytical tools, reflecting a field oriented toward improving clinical outcomes and reducing re-injury risk.

Collectively, these findings highlight the growing importance of interdisciplinary and athlete-centered approaches in ACL rehabilitation. This study identifies leading contributors, major research themes, and emerging priorities, offering clinicians, rehabilitation specialists, and researchers a structured evidence map to inform future investigation, support RTS decision-making, and guide efforts to improve functional recovery and long-term athletic participation following ACL injury.

## Figures and Tables

**Table 1:** Most important source in RTS after ACL injury research.

Rank	Journal	TP	Publisher	SCY	CS	SNIP
1	*Orthopaedic Journal of Sports Medicine*	175	SAGE	2013	4.5	1.278
2	*Knee Surgery Sports Traumatology Arthroscopy*	137	John Wiley & Sons	1993	8.4	1.689
3	*American Journal of Sports Medicine* ^1^	120	SAGE	1973	8.8	2.039
4	*Physical Therapy in Sport*	55	Elsevier	2000	4.8	1.192
5	*Knee*	49	Elsevier	1994	3.7	0.999
6	*Arthroscopy Journal of Arthroscopic and Related Surgery*	43	John Wiley & Sons	1985	9.6	1.887
7	*Sports Health*	43	SAGE	2009	6.6	1.793
8	*International Journal of Sports Physical Therapy*	37	North American Sports Medicine Institute	2019	3.1	1.093
9	*Journal of Experimental Orthopaedics*	25	John Wiley & Sons	2014	3.3	1.051
10	*Arthroscopy Sports Medicine and Rehabilitation*	23	John Wiley & Sons	2019	3.6	0.956

Note: Source: Scopus; TP: Total Publications; SCY: Scopus Coverage Years till 2026; CS = CiteScore 2024; SNIP = Source Normalized Impact per Paper; details are as of April 2026. ^1^ Formerly known as: *The Journal of Sports Medicine*.

**Table 2:** Top impactful works based on the total number of citations.

Rank	Title	Source Title	TC ^1^
1	Biomechanical measures during landing and postural stability predict second anterior cruciate ligament injury after anterior cruciate ligament reconstruction and return to sport [63]	*American Journal of Sports Medicine*	1125
2	Simple decision rules can reduce reinjury risk by 84% after ACL reconstruction: The Delaware-Oslo ACL cohort study [64]	*British Journal of Sports Medicine*	1060
3	Likelihood of ACL graft rupture: Not meeting six clinical discharge criteria before return to sport is associated with a four times greater risk of rupture [19]	*British Journal of Sports Medicine*	691
4	Return to the preinjury level of competitive sport after anterior cruciate ligament reconstruction surgery: Two-thirds of patients have not returned by 12 months after surgery [65]	*American Journal of Sports Medicine*	526
5	Psychological responses matter in returning to preinjury level of sport after anterior cruciate ligament reconstruction surgery [66]	*American Journal of Sports Medicine*	473
6	Anterolateral Ligament Reconstruction Is Associated with Significantly Reduced ACL Graft Rupture Rates at a Minimum Follow-up of 2 Years: A Prospective Comparative Study of 502 Patients from the SANTI Study Group [67]	*American Journal of Sports Medicine*	456
7	Incidence of contralateral and ipsilateral Anterior Cruciate Ligament (ACL) injury after primary ACL reconstruction and return to sport [68]	*Clinical Journal of Sport Medicine*	456
8	Muscle strength and hop performance criteria prior to return to sport after ACL reconstruction [69]	*Knee Surgery, Sports Traumatology, Arthroscopy*	383
9	The impact of quadriceps femoris strength asymmetry on functional performance at return to sport following anterior cruciate ligament reconstruction [70]	*Journal of Orthopaedic and Sports Physical Therapy*	376
10	The impact of psychological readiness to return to sport and recreational activities after anterior cruciate ligament reconstruction [71]	*British Journal of Sports Medicine*	369

Source: Scopus; TC: Total Citations Received; details are as of April 2026. ^1^ Formerly known as: *The Journal of Sports Medicine*.

**Table 3:** Leading authors in the RTS after ACL injury research.

Rank	Authors	TP
1	Webster, K.E.	47
2	Paterno, M.V.	43
3	Feller, J.A.	39
4	Hewett, T.E.	38
5	Schmitt, L.C.	36
6	Hamrin Senorski, E.	31
7	Snyder-Mackler, L.	31
8	Thomeé, R.	31
9	Samuelsson, K.	28
10	Piussi, R.	26

Source: Scopus; TP: Total Publications; details are as of April 2026.

**Table 4:** Primary themes, patterns, and emerging areas within the research topic.

Theme	Keywords
Basic Themes	Return to sport, anterior cruciate ligament, anterior cruciate ligament reconstruction, football (soccer), knee injury, anterior cruciate ligament injury, athlete, re-injury, anterior cruciate ligament rupture, patient reported outcome, pediatric, knee function, fear of re-injury, sports medicine, psychology, sports injury, muscle strength, female athlete, functional test, kinesiophobia, patient reported outcome measures, psychological factors, performance, youth athlete, anterior cruciate ligament repair, graft rupture, basketball, isokinetic test, national football league, test battery, women, knee joint, prevention, professional athlete, athletic performance, isokinetic strength, psychological, sports performance, exercise, functional performance, ligament reconstruction, motion analysis, national basketball association, skeletal immaturity, fear, athletic injury, muscle function, neuromuscular training, prognosis, sports participation, Australian football, psychosocial factors, quadriceps tendon autograft, readiness, return to performance, subsequent injury, revision, lateral extra-articular tenodesis, arthroscopy, functional outcome, meniscus, graft, graft failure, bone patellar tendon bone, bone patellar tendon bone autograft, complication, paediatric, all-inside, anterior cruciate ligament revision, bone-patellar tendon-bone graft, failure, hamstring tendon autograft, patellar tendon, psychological readiness, anterior cruciate ligament-return to sport after injury scale, hop test, limb asymmetry, quadriceps strength, strength, anterior cruciate ligament tear, second injury, single leg hop test, strength test, IKDC, landing mechanics, vertical jump, outcome measure, symmetry, return to sport criteria, clinical outcome, hamstring autograft, re-tear, sex, quadriceps autograft, rehabilitation, knee, physiotherapy, sports, injury, ligament, knee ligament, surgery, orthopaedic, isokinetic, knee surgery, running, osteoarthritis, psychological aspects of sport, survey, physical therapy modalities, rupture, athletic training, functional assessment, rugby, adolescent athlete, outcome, elite athlete, repair, cartilage, let, prehabilitation
Niche Themes	Pivoting sports, postural stability, gender, jump landing, validity, reliability, psychological patient-reported outcome measure (PROM), questionnaire
Emerging Themes	Allograft, magnetic resonance imaging, autograft, graft maturation, lars, signal-to-noise quotient, sports activity, physical activity, quality of life, accelerometer, return to work, double bundle, pivot shift, ACL outcomes, ACL re-injury, knee rehabilitation, confidence, psychological response, re-rupture, qualitative research, qualitative, anterior cruciate ligament surgery, post-operative rehabilitation, kinematics, kinetics, landing, weakness, anterolateral ligament, anterolateral ligament reconstruction, anterior cruciate ligament rehabilitation, revision ACL reconstruction
Motor	Biomechanics, injury prevention, quadriceps, hamstring, return to sport testing, asymmetry, proprioception, movement system, limb symmetry, gait analysis, machine learning, neuromuscular control, pediatric sports medicine, electromyography, isokinetic dynamometer, lower extremity, movement analysis, postural balance, quadriceps muscle, risk factors, contralateral, criteria, function, internal brace, jumping, medial collateral ligament, multi-ligament knee injury, multi-ligament knee reconstruction, posterolateral corner, systematic review

Note: IKDC = International Knee Documentation Committee score.

**Table 5:** Evolution and frequency of research themes in RTS after ACL injury research from around 2016 to 2026.

Period	Dominant Keywords	Thematic Focus & Research Emphasis
2016–2018	Psychological, movement analysis, muscle function, limb symmetry, athletic injury	Early research focused on foundational biomechanical and neuromuscular deficits, particularly movement analysis, limb symmetry, and muscle function. The inclusion of psychological aspects reflects the initial recognition of mental factors in rehabilitation.
2018–2020	Single-leg hop test, outcome, knee function, quadriceps strength, second injury	Research transitioned toward functional performance assessment, emphasizing hop tests, quadriceps strength, and objective outcome measures. Increased attention to second injury risk emerged during this phase.
2020–2022	ACL reconstruction, return to sport, sports, pediatric, knee function	A consolidation phase integrating clinical and sports perspectives. Studies explored RTS timelines, pediatric populations, and surgical outcomes, reflecting broader sports medicine integration.
2022–2024	Lateral extra-articular tenodesis, PROMs, physiotherapy, muscle strength, sports medicine	This period emphasized surgical innovation combined with patient-centered outcomes. The use of PROMs and physiotherapy indicates a shift toward functional recovery quality and rehabilitation effectiveness.
2024–2026	Psychological readiness, physiotherapy, hamstring autograft, MRI, football (soccer), ACL rupture	Recent trends reveal a multidimensional and precision-based approach, integrating psychological readiness, advanced imaging (MRI), sport-specific contexts, and surgical refinements (autografts, tenodesis). This phase reflects personalized rehabilitation, performance optimization, and injury risk management.

Note: RTS = return to sport; PROMs = patient-reported outcome measures; MRI = Magnetic resonance imaging.

**Table 6:** Common themes in recent research on RTS after ACL injury (2021–2026).

Sl. No	Theme	Representative Keywords
1	Psychological and Neurocognitive Readiness	Confidence, fear of re-injury, psychological readiness, motivation, emotions, sports and exercise psychology, neurocognitive training
2	Rehabilitation and Performance Optimization	Rehabilitation, physiotherapy, recovery, blood flow restriction training, performance, proprioception, and isokinetic evaluation
3	Biomechanical and Functional Recovery	Gait symmetry, limb asymmetry, muscle strength, quadriceps femoris, ground reaction force, countermovement jump, functional capacity
4	Surgical and Structural Innovation	Hamstring autograft, quadriceps autograft, anterolateral ligament, lateral extra-articular tenodesis, multi-ligament knee reconstruction, meniscectomy, meniscus injury, knee dislocation, sports surgery
5	Clinical and Outcome Validation	Clinical outcome, PROMs, patient-accepted symptom status, validity, sports medicine
6	Technological and Analytical Advancements	MRI, machine learning, second ACL injury, second knee injury

Note: PROMs = Patient-reported outcome measures; MRI = Magnetic resonance imaging.

## Data Availability

The Scopus-derived bibliographic dataset, the standardized keyword thesaurus file, and the VOSviewer and Biblioshiny analysis parameters used in this study are available from the corresponding author upon reasonable request.

## Supplementary Materials

The following supporting information can be downloaded at https://www.mdpi.com/article/10.3390/healthcare14142099/s1, Table S1: Search and Screening Strategy; Supplementary Materials S2: Search Query.
