# Supplementary material for: Return to Sport After Anterior Cruciate Ligament Injury: A Scopus-Based Bibliometric Analysis
Source: Healthcare (Basel). 2026 Jul 14;14(14):2099. doi: 10.3390/healthcare14142099 (PMC13412021; doi:10.3390/healthcare14142099)
Supplement: Supplementary file 1 [file healthcare-14-02099-s001.zip › healthcare-4387589-supplementary.pdf]

Return to Sport After Anterior Cruciate Ligament Injury: A Scopus-Based Bibliometric Analysis of Rehabilitation Research in Athletes

Supplementary Materials S1

Table S1. Search and Screening Strategy

Search	Theme	Keywords	Search on	Documents
1	ACL	"anterior cruciate ligament*" OR "ACL"	Title OR Keywords	44,895
2	Return to sport	"return to play*" OR "return-to-sport*" OR "return to sport*" OR "back to sport*" OR "return-to-competition*" OR "return to competition*"	Title OR Keywords	10,283
3	ACL AND Return to Sport [Search 1 AND 2]			2,742
4	In Article (Other)			2,063
5	In English			2,024
6	Expert Screening 656			1368

Source: Scopus; Date of Search 4 April 2026.

Supplementary Materials S2. Search Query

((TITLE ("return to play" OR "return-to-sport*" OR "return to sport*" OR "back to sport*" OR "return-to-competition*" OR "return to competition*") OR KEY ("return to play" OR "return-to-sport*" OR "return to sport*" OR "back to sport*" OR "return-to-competition*" OR "return to competition*"))) AND ((TITLE ("anterior cruciate ligament*" OR "ACL") OR KEY ("anterior cruciate ligament*" OR "ACL"))) AND (LIMIT-TO (LANGUAGE , "English")) AND (LIMIT-TO (DOCTYPE , "ar"))
